# Gut health and physiological aspects of broiler chicken fed zeolite as a dietary supplement: its effect on growth, cecal microbiota and digesta viscosity, digestive enzymes, carcass traits, blood constituents and antioxidant parameters

**DOI:** 10.1186/s12917-025-05006-1

**Published:** 2025-09-09

**Authors:** Ibrahim A. Abdel-Kader, Shaaban Saad Elnesr, Bothaina Y. Mahmoud, Ensaf A. El-Full, Ahmed M. Emam

**Affiliations:** Department of Poultry Production, Faculty of Agriculture, Fayoum University, Fayoum, 63514 Egypt

**Keywords:** Gut health, Zeolite, Supplementation, Physiology, Broiler

## Abstract

This study investigated the impact of dietary zeolite supplementation on growth, cecal microbiota and digesta viscosity, digestive enzymes, carcass traits, blood constituents, and antioxidant parameters of broilers. A completely randomized design was used with 240 one-day-old broiler chicks randomly assigned to three dietary treatments (0%, 1.5%, and 3% zeolite as a feed additive) with four replicates of 20 chicks each. Data were analyzed using one-way ANOVA followed by Duncan’s multiple range test (*p* < 0.05). The findings indicated that growth performance was enhanced by the addition of zeolite, especially at the 3% supplementation level. Both blood biochemistry and hematological parameters showed significant improvement (*p* < 0.05) with supplementation at both 3% and 1.5% zeolite levels. Antioxidant parameters indicated significantly elevated serum amylase activity and total antioxidant capacity with both 3% and 1.5% zeolite diets. Cecal microbiota was improved by zeolite, enhancing beneficial bacteria and reducing *E. coli* and anaerobes. Carcass characteristics were positively influenced by 3% zeolite. Digesta viscosity and intestinal pH decreased with zeolite inclusion. In conclusion, the inclusion of zeolite up to 3% in broiler diets had positive effects on diverse aspects of health, intestinal microbiota, performance, and blood constituents.

## Introduction

One of the primary factors influencing a bird’s performance and, consequently, its financial yield in the poultry industry is intestinal health. Numerous studies have examined the use of natural dietary supplements to enhance intestinal health [1, 2]. Using natural feed additives in poultry nutrition is a widespread tradition for improving physiological indices, enhancing productivity, health, and product quality [3, 4]. Natural zeolites are sedimentary volcanogenic rocks. The structural composition is formed mainly from a tetrahedral framework of aluminosilicate [5], which results in a net negative charge. The additional cations such as Na^+^, K^+^, Ca^2+^, and Mg^2+^, are exchangeable ions and balance the negative charge [5]. Natural zeolite is characterized by its availability, high porosity and large surface area, which make it suitable as an ion exchanger and absorbent for numerous applications [5]. To take advantage of these natural zeolite features, its use as an adsorbent for gases, pollutants, toxins, water and ion-exchangeable materials has been developed [6].

In poultry production, many studies have addressed the use of zeolite either as a feed additive [7] or as an inclusion in the litter [8]. The inclusion of natural zeolite in poultry litter accompanied by desirable findings such as improvement of the internal environmental conditions [8], reduction of emissions such as ammonia and odor [9], and enhancement of manure properties in the poultry house. Fortunately, these findings have shown promising effects on the productive performance, physiological status, and poultry health, in addition to improving indoor air quality and reducing harmful emissions, ammonia emissions, which subsequently prevents their [8, 9]. Supplementation of clay minerals in diets can reduce pollution and enhance the pH of litter, nitrogen digestibility, and growth performance in poultry [10].

As a novel, available and inexpensive feed additive, several investigators have tested zeolite, and found significant enhancement in productive performance [11–13]. Moreover, zeolite improved enzymatic activity, digestibility, absorption and the health of gut, consequently improving digestive function and feed efficiency [14–16]. Interestingly, zeolite can enhance villus development, modify gut microbiota structure [17], act similar to antibiotics against harmful intestinal gram- negative bacteria [18], increase antioxidant capacity [19], absorb harmful substances in the gut, and reduce the toxicity of aflatoxin [20] in poultry. Based on the above, it is hypothesized that the inclusion of zeolite in the diet as a feed additive is expected to exert favorable influences on broiler chicks. Although the effects of zeolite on broiler chickens have been studied in the past, these studies did not cover all parameters that are considered important aspects of gut health and nutrient absorption. Accordingly, this study was designed to determine the influences of dietary natural zeolite on growth performance, some blood constituents, iron profile, blood ammonia, digestive enzymes, intestinal microbiota and viscosity, antioxidant indices, and carcass traits of broiler chicks. The present study allows for a more comprehensive understanding of zeolite’s multifaceted role as a functional feed additive in poultry nutrition.

## Materials and methods

### Ethics and consent to participate

All animal-based experimental procedures conducted in the current study were approved by Fayoum University Institutional Animal Care and Use Committee (FU-IACUC), Egypt (Approval Code No. AEC2341).

### Source of the animals

Broiler chicks (Arbor Acres) were obtained from a local commercial hatchery and used in this experiment.

### Birds, diets and experimental design

Total of 240 unsexed broiler chicks (Arbor Acres) at one-day-old were randomly distributed with equal body weights (46.20 g ± 0.14 g) into three groups, each consisting of four replicates, with 20 chicks per replicate (pen): control and two treatments, which consisted of the control diet plus 1.5% and 3% zeolite, respectively. Natural zeolite (Ca, K_2_, Mg)^4^ (Al_8_ Si_40_) O_96_·24H_2_O has a cation exchange capacity of 1.50 mol/kg and has high affinity and selectivity for NH^4+^ ions. Zeolite was purchased from A & O Trading, Giza, Egypt.

Chicks were reared in a semi-closed house on floor pens with straw litter. Each pen had a total floor area of 2 m², accommodating 20 birds per pen. Each pen was equipped with 2 feeders and 2 drinkers to ensure adequate feeding and watering space. The lighting schedule was 23 h of light: 1 h of dark daily (from 1 to 7 days), while the lighting schedule was 18 h of light, 6 h of dark daily (from 8 to 35 days) [21]. Temperature was maintained at 32 °C from 0 to 7 days and progressively reduced to reach 20 °C by 35 days of age, with an average relative humidity of 60–65%.

Three maize-soybean meal basal diets (starter 1–10 days, grower 11–24 days and finisher 25–35 days) were formulated to deliver adequate nutrients for chicks according to the broiler management guide. Table 1 exhibited the ingredients and chemical composition of the basal diets. Feed in mash form and water were provided *ad libitum*.

**Table 1:** Ingredients and chemical composition of the basal diets

Ingredients	Starter diet		Grower diet	Finisher diet
	From 1: 10 days of age		From 11: 24 days of age	From 25: 35 days of age
Yellow corn	60.40		65.00	69.50
Soybean meal (46% CP)	25.00		25.00	14.15
Corn gluten (60% CP)	9.64		5.70	10.00
Di-calcium phosphate	2.04		1.65	1.65
CaCO3	1.20		1.00	1.00
NaCl	0.30		0.30	0.30
Vitamin and mineral premix^a^	0.30		0.30	0.30
Lysine	0.51		0.50	0.56
Methionine	0.16		0.15	0.14
Soybean oil	0.45		0.40	2.40
Total	100.00		100.00	100.00
Calculated analysis				
Crude protein (CP) %	22.97	20.98		18.99
ME (kcal/kg diet)	2998	3002		3209
Ether extract%	3.19	3.21		5.40
Crude fiber%	3.27	3.29		2.72
Calcium%	0.99	0.83		0.80
Available Phosphorus %	0.50	0.43		0.42
Methionine%	0.55	0.51		0.49
Lysine *%*	1.33	1.29		1.10

*ME* Metabolizable energy

^a﻿^ Each 3.0 Kg of the broiler Vit. and Min. premix contains: Vit. A 12,000,000 IU, Vit. D_3_ 2,000,000 IU, Vit. E 40,000 mg, Vit. K_3_ 4000 mg, Vit. B_1_ 3000 mg, Vit. B_2_ 6000 mg, Vit. B_6_ 4000 mg, Vit. B_12_ 30 mg, choline chloride 350,000 mg, biotin 80 mg, folic acid 1500 mg, nicotinic acid 30 g, niacin 30,000 mg, pantothenate acid 12,000 mg, Zn 70,000 mg, Cu 4 g, Fe 40,000 mg, Co 10,000 mg, Se 200 mg, I 300 mg, Mn 70,000 mg and completed to 3.0 Kg by calcium carbonate

### Growth performance

Live body weight (LBW) of birds was individually weighed at 1 and 35 days of age. Feed intake (FI) per pen was recorded throughout the test period (from 1 to 35 days). Body weight gain (BWG) was calculated as the difference between final and initial body weights. Feed conversion ratio (FCR) was determined as the ratio of feed intake to weight gain. The European Production Efficiency Factor (EPEF) at day 35 was calculated using the following formula:

$$\mathrm{EPEF}=\left[\mathrm{final}\;\mathrm{BW}\times\mathrm{survival}\;\mathrm{rate}\%/\mathrm{FCR}\times35\right]\times10$$

The growth rate (GR_1−35_%) was calculated using the following formula:

$${\mathrm{GR}}_{1-35}=\left[\left({\mathrm{BW}}_{35^-}{\mathrm{BW}}_1\right)\times100\right]/\left[0.5\left({\mathrm{BW}}_{35}+{\mathrm{BW}}_1\right)\right]$$

### Carcass traits

On day 35, eight birds from each group (*n* = 24) were slaughtered by cutting the jugular vein, then defeathered and eviscerated. Carcass traits (carcass%, gizzard%, liver%, heart%, thigh bone%, breast bone%) were calculated relative to LBW [22]. Thigh bones include the femur, the ilium, ischium, tibia, and fibula. Breast bones include scapula, coracoid, clavicle, sternum, and humerus.

### Digesta pH and viscosity

At 35 days of age, the entire gastrointestinal tract was immediately removed for visual evaluation and digesta sampling. The digesta from the duodenum, ileum and jejunum was collected into separate tubes, and was vortexed to obtain a homogenous content from each anatomical part of the intestine/chick. The viscosity of intestinal digesta was determined using a Brookfield DV-II + PRO Digital Viscometer (Brookfield Engineering Laboratories, Stoughton, MA, USA) according to Tsiouris et al. [23]. The pH of the duodenum, ileum and jejunum from each bird was measured using a digital pH-meter (pH 315i, WTW Wissenschaftlich-TechnischeWerkstätten, Weilheim, Germany).

### Cecal microbiota analysis

Eight samples per treatment were collected from both cecal pouches of each bird within groups. Total anaerobes, *Escherichia coli* and *Lactobacillus* were counted [24]. The findings were expressed as log10 colony-forming units per gram of digested intestinal content.

### Biochemical analyses

On day 35, blood samples were collected from the slaughtered chicks (*n* = 24, 8 in each group) by cutting the jugular vein. Each sample was divided into two tubes: The 1 st tube (without an anticoagulant) was centrifuged for 15 min (755 *g* value) to separate the serum that was stored at − 20◦C until the laboratory analyses. The 2nd tube contained the whole blood with an anticoagulant to determine haematological parameters (hematocrit, hemoglobin, red blood cells (RBCs), and white blood cells (WBCs)).

### Blood biochemistry

Serum biochemical parameters (creatinine, uric acid, albumin, total protein, iron, total iron-binding capacity (TIBC), aspartate aminotransferase (AST), alanine aminotransferase (ALT), ammonia and total bilirubin levels) were determined colorimetrically using appropriate commercial diagnostic kits produced by Abcam Inc, UK. Amylase and lipase enzymes were assayed by Friedman and Young [25]. The trypsin enzyme was determined using the Bovine Trypsin ELISA Kit MBS706461 according to Zhou et al. [26].

### Antioxidant parameters

Total antioxidant capacity (T-AOC) and superoxide dismutase (SOD) in the liver and serum using commercial kits were analyzed colorimetrically using a commercial kit and an Infinite F50 spectrophotometer (Tecan Trading AG, Männedorf, Switzerland). For liver analysis,, the appropriate amount of sample was prepared and homogenized according to the manufacturer’s instructions. Catalogue numbers of the kits used: T-AOC (E-BC-K136-M) and SOD (E-BC-K019-S), both from Elabscience, United States).

### Statistical analysis

All data were tested for normality using the Shapiro-Wilk test (*p* > 0.05) and homogeneity of variance using Levene’s test (*p* > 0.05). The recorded data were statistically analyzed by one-way analysis of variance (ANOVA) using SAS [27] to calculate the treatment specific means using the following model :

$${\mathrm Y}_{\mathrm{ij}}=\mathrm\mu+{\mathrm Z}_{\mathrm i}+{\mathrm e}_{\mathrm{ij}}$$

Y_ij_= Observation of the variable measured. µ = Overall mean. Z_i_= Effect of zeolite treatment, e_ij_= Experimental error. Means of treatment were compared using Duncan’s multiple range test. A priori power analysis using GPower software demonstrated adequate statistical power (0.82) for detecting treatment differences.

## Results

### Growth performance

Least square means of body weight (BW_35_), body weight gain (BWG_1−35_), growth rate % (GR_1−35_%), feed intake (FI_1−35_), feed conversion ratio (FCR_1−35_), and EPEF as affected by dietary supplementation of zeolite are shown in Table 2. Except for BW_1_, the inclusion of zeolite in broiler diets significantly improved (*P* < 0.0001) all studied growth traits. Chicks fed 3% zeolite had the heaviest BW_35_, the highest BWG_1−35_, and EPEF, the lowest FI_1−35_, the higher GR_1−35_% and the better FCR_1−35_ followed by those fed 1.5% zeolite and the control group, respectively. Compared with the control, the zeolite supplementation at a level of 3% proportionally improved for LBW_35_, BWG_1−35_, GR_1−35_%, FCR_1−35_ and EPEF (+ 4.365%, + 4.452%, + 0.172%, −5.611% and + 12.223%, respectively). The results for LBW_35_, BWG_1−35_, GR_1−35_%, FCR_1−35_ and EPEF were in favoring the 3% level of zeolite supplementation which was superior to the 1.5% level by + 1.475%, + 1.505%, + 0.057%, − 1.911% and + 3.403%, respectively.

**Table 2:** Effect of dietary zeolite supplementation on productive performance of broiler chicks

Items	LBW_1_ (g)	LBW_35_ (g)	BWG_1−35_ (g)	GR_1−35_%	FI_1−35_ (g)	FCR_1−35_ (g/g)	EPEF
Control	46.150	2313.125^c^	2266.975^c^	192.150^b^	3382.450^a^	1.497^a^	435.675^c^
Zeolite 1.5%	46.213	2379.000^b^	2332.788^b^	192.370^a^	3291.973^b^	1.413^b^	472.828^b^
Zeolite 3.0%	46.225	2414.103^a^	2367.897^a^	192.482^a^	3279.165^c^	1.386^b^	488.921^a^
SEM	0.137	10.967	11.059	0.034	3.217	0.007	4.347
^1^Zeolite1.5%/control (%)	-	102.848	102.903	100.114	97.325	94.389	108.529
^2^Zeolite3%/control (%)	-	104.365	104.452	100.172	96.947	92.585	112.223
^3^Zeolite3%/Zeolite1.5% (%)	-	101.475	101.505	100.057	99.611	98.089	103.403
Probability	0.9193	0.0001	0.0001	0.0001	0.0001	0.0001	0.0001

^a, b,c^: Means in the same column followed by different letters are significantly different at (*p* ≤ 0.05)

*LBW*_1_ Live body weight at 1 day of age, *LBW*_35_ Live body weight at 35 days of age, *BWG*_1−35_ Body weight gain from 1 to 35 days of age, *GR*_1−35_% Growth rate during 1–35 days of age,*FI*_1−35_ Feed intake during 1–35 days of age,* FCR*_1−35_ Feed conversion ratio during 1–35 days of age, *EPEF* European production efficiency factor at day 35, *SEM* Standard error of mean

^1^Values are expressed relative to control = (1.5% zeolite treatment mean/control mean) *100

^2^Values are expressed relative to control = (3% zeolite treatment mean/control mean) *100

^3^Values are expressed relative to 1.5% zeolite treatment = (3% zeolite treatment mean/1.5% zeolite treatment mean) *100

### Intestinal pH, viscosity and microbiota

Treatment effects on intestinal pH and viscosity are presented in Table 3. Except for cecal pH, intestinal pH significantly decreased (*p* < 0.05) with zeolite inclusion in broiler diets. Zeolite inclusion in the diet at levels of 1.5 to 3%, significantly decreased viscosity in all studied intestinal segments.

**Table 3:** Effect of dietary zeolite supplementation on intestinal pH and viscosity and cecal microflora counts of broiler chicks

Items	pH				Viscosity (millipascal second)			Cecal microflora counts (log 10 CFU/g)		
	Duodenum	Jejunum	Ileum	Cecum	Duodenum	Jejunum	Ileum	Lactobacillus sp	E. coli	Total anaerobes count
Control	5.13^a^	5.60^a^	6.44^a^	6.87	2.50^a^	2.94^a^	3.68^a^	8.12^c^	7.44^a^	9.21^a^
Zeolite 1.5%	4.96^ab^	5.21^b^	6.10^b^	7.01	2.00^b^	2.44^b^	2.81^b^	8.36^b^	6.38^b^	7.55^b^
Zeolite 3.0%	4.89^b^	5.19^b^	6.31^ab^	6.82	1.81^c^	2.05^c^	2.48^c^	8.82^a^	5.89^c^	7.18^c^
SEM	0.06	0.08	0.09	0.12	0.06	0.05	0.07	0.11	0.11	0.10
Probability	0.0468	0.0017	0.0442	0.5339	0.0001	0.0001	0.0001	0.0012	0.0001	0.0001

^a, b,c^: Means in the same column followed by different letters are significantly different at (*p* ≤ 0.05)

SEM Standard error of mean

Increasing levels of zeolite supplementation in broiler diets were significantly increased *Lactobacillus spp.* and decreased the counts of *E. coli* and total anaerobe counts (Table 3). The diet supplemented with 3% zeolite desirably had the highest counts of beneficial microbiota (*Lactobacillus sp*) (*p* = 0.0012) and the lowest *E. coli* and total anaerobes counts (*p* = 0.0001) compared with the birds in the other groups (1.5% zeolite and the control).

### Digestive enzymes

The results of digestive enzyme activities are summarized in Table 4. The findings indicated that the addition of dietary zeolite led to a significant increase (*p* < 0.05) in amylase activity. However, the activities of lipase and trypsin showed insignificant increase (*p* > 0.05) as the dietary zeolite levels were elevated.

**Table 4:** Effect of dietary zeolite supplementation on digestive enzymes of broiler chicks

Items	Amylase U/L	Lipase U/L	Trypsin U/L
Control	311.88^b^	21.63	26.06
Zeolite 1.5%	395.63^a^	23.25	27.88
Zeolite 3.0%	398.13^a^	23.00	27.71
SEM	11.73	0.65	0.57
Probability	0.0001	0.1895	0.0670

^a, b^: Means in the same column followed by different letters are significantly different at (*p* ≤ 0.05)

*SEM* Standard error of mean

### Carcass characteristics

According to Table 5, there is no statistical difference in the carcass%, liver%, heart%, thigh bone%, breast bone%. On the other hand, diets containing 3% zeolite resulted in significantly higher (*p* < 0.05) gizzard%, and breast bone% compared to other treatments.

**Table 5:** Effect of dietary zeolite supplementation on carcass characteristics of broiler chicks

Items	Carcass%	Gizzard%	Liver%	Heart%	Thigh bone%	Breast bone%
Control	72.95	2.22^b^	2.63	0.68	10.54	12.82
Zeolite 1.5%	74.60	2.58^a^	2.72	0.70	10.26	12.94
Zeolite 3.0%	75.01	2.59^a^	2.80	0.71	10.72	12.82
SEM	0.70	0.06	0.06	0.03	0.39	0.48
Probability	0.1110	0.0004	0.1460	0.7504	0.7133	0. 9794

^a, b^: Means in the same column followed by different letters are significantly different at (*p* ≤ 0.05)

*SEM* Standard error of mean

### Blood biochemistry

AST, total protein, albumin and calcium levels in blood were significantly increased with increasing zeolite in the diet as shown in Table 6. The group fed the diet supplemented with 3% zeolite had higher values than the other groups. On the contrary, levels of blood ammonia, uric acid and creatinine were significantly decreased with the dietary zeolite supplementation where the control showed the highest levels of these parameters. On the other hand, the birds fed 1.5% zeolite had significantly the highest blood phosphorus and lower total bilirubin levels compared with the other groups, whereas the addition of zeolite in the broiler diet resulted in insignificant increases in ALT and globulin levels.

**Table 6:** Effect of dietary zeolite supplementation on blood biochemical parameters of broiler chicks

Items	Blood amonia (mmol/L)	ALT (U/L)	AST (U/L)	Uric acid (mg/dL)	Creatinine (mg/dl)	Total protein (g/dl)	Albumin (g/dl)	Globulin (g/dl)	Total bilirubin level (mg/dl)	Calcium (mg/dL)	Phosphorus (mg/dL)
Control	0.32^a^	5.91	176.81^b^	5.05^a^	0.45^a^	3.24^b^	1.13^b^	2.11	0.93^a^	9.86^c^	7.60^b^
Zeolite 1.5%	0.22^b^	6.07	184.53^a^	4.48^b^	0.38^b^	3.79^a^	1.56^a^	2.23	0.76^b^	10.06^b^	8.84^a^
Zeolite 3.0%	0.22^b^	6.37	185.91^a^	4.30^b^	0.37^b^	3.87^a^	1.61^a^	2.27	0.77^b^	10.23^a^	7.52^b^
SEM	0.01	0.17	2.16	0.14	0.02	0.07	0.04	0.07	0.03	0.04	0.08
Probability	0.0001	0.1520	0.0150	0.0025	0.0063	0.0001	0.0001	0.2434	0.0007	0.0001	0.0001

^a, b,c^: Means in the same column followed by different letters are significantly different at (*p* ≤ 0.05)

*SEM* Standard error of mean, *AST* Aspartate aminotransferase, *ALT* Alanine aminotransferase

### Hematological parameters and iron profile

Zeolite supplementation in the diet had a significant impact (*p* < 0.05) on hematological parameters except for WBCs as shown in Table 7. The inclusion of zeolite in broiler diets was significantly accompanied by increases in Hb, RBCs, HCT%, and serum iron and a decrease in TIBC compared with the the control.

**Table 7:** Effect of dietary zeolite supplementation on hematological parameters and iron profile of broiler chicks

Items	Hemoglobin (g/dl)	Red blood cell (10^6^/mm^3^)	White blood cell (10^3^/mm^3^)	Hematocrit (HCT) %	Serum iron (mg/dL)	Serum TIBC (mg/L)
Control	11.11^b^	3.27^b^	24.67	38.30^b^	0.26^c^	0.48^a^
Zeolite 1.5%	12.30^a^	3.60^a^	24.82	42.48^a^	0.30^b^	0.40^b^
Zeolite 3.0%	12.27^a^	3.68^a^	24.83	42.81^a^	0.31^a^	0.40^b^
SEM	0.18	0.10	0.26	0.58	0.004	0.01
Probability	0.0002	0.0257	0.8915	0.0001	0.0001	0.0001

^a, b,c^: Means in the same column followed by different letters are significantly different at (*p* ≤ 0.05)

*SEM* Standard error of mean, *TIBC* total iron-binding capacity

### Antioxidant parameters

The findings of serum and liver antioxidant indices, such as T-AOC and SOD levels as affected by zeolite are presented in Table 8. The dietary supplementation with zeolite in the broiler diet did not have a significant impact on any of the following: serum SOD, liver T-AOC or liver SOD. However, chicks fed both 3% and 1.5% zeolite diets had significantly higher (*p* < 0.05) levels of serum T-AOC, and liver T-AOC than the control group.

**Table 8:** Effect of dietary zeolite supplementation on serum and liver antioxidant indices of broiler chicks

Items	Serum		Liver	
	T-AOC (U/mL)	SOD (U/mL)	T-AOC (U/mg/protein)	SOD (U/mg/protein)
Control	12.26^b^	170.97	7.00^b^	166.35
Zeolite 1.5%	13.82^a^	172.10	7.61^a^	169.91
Zeolite 3.0%	13.84^a^	172.61	7.60^a^	169.21
SEM	0.30	1.06	0.18	1.02
Probability	0.0016	0.5425	0.0509	0.0524

^a, b,c^: Means in the same column followed by different letters are significantly different at (*p* ≤ 0.05)

*SEM* Standard error of mean, *T-AOC* Total antioxidant capacity, *SOD* Superoxide dismutase

## Discussion

Zeolite has garnered growing interest as a potentially sustainable approach to enhancing poultry health, optimizing feeding practices, and contributing to a more environmentally conscious approach. Furthermore, there is a rising consumer demand for food originating from chemical-free production systems. Over time, poultry producers will need to avoid using pharmaceutical or chemical feed additives and instead depend on natural substances. The supplementation of dietary zeolite has exhibited significant improvement in the growth performance of broilers. Similar findings were reported by Emam et al. [12] and AL-musawy et al. [13]. There are two explanations regarding the fundamental mechanisms of zeolites: Firstly, the impact of ammonia binding, which results in the removal of toxic effects induced by NH_4_^+^ originating from microbial activity in the intestine [28]. Secondly, the slowing of digesta transit leads to more efficient utilization of nutrients [29]. Furthermore, the favorable impact of zeolite on animal growth depends on its composition, featuring an extensive array of vital macro- and micro-chemical elements crucial for supporting animal development. These elements exist in an ionic state and have the potential to be released into organisms. Additionally, these constituents have the ability to counteract the adverse influences of existing toxic substances, for instance hydrogen sulfide and ammonia gas, while also maintaining low levels of bacterial contamination in the intestine, including microbes like *Salmonella*, *E. coli*, and dysentery bacilli, owing to their remarkable absorption capacity. Consequently, this absorption process facilitates the removal of harmful substances from the animals’ bodies [30]. The enhancement of productive performance is intricately related to the alterations that occur during nutrient digestion and the digestive enzyme secretion [31]. This enhancement could, in turn, improve nutrient digestion and absorption, reflecting an improvement in the productive performance, and the effective immobilization of enzymes as well as positive impacts on the gut microbiota of birds [17]. Prasai et al. [32] investigated the impacts of supplementing broiler chicken diets with different levels of zeolite (1%, 2%, and 4%). They found that the enhancements in FCR and body weight of broiler birds were possibly related to improved villi function and enhanced gut health. On the other hand, Abdelrahman et al. [7] stated that the addition of dietary zeolite did not lead to any significant improvements in the feed efficiency and growth performance of broiler chicks.

The impact of zeolites on feed efficiency is believed to arise from multiple factors, including a potential slowdown in food transit through the gut, enzyme immobilization, and modulation of gut microbiota [33]. The potential enhancement in nutrient utilization might be linked to shifts in the composition or distribution of gastrointestinal microbiota. Correspondingly, Olver [34] reported significant reductions in colony counts within both proximal and distal intestinal regions when zeolites were incorporated into diets. Zeolites could potentially aid in promoting better blood circulation within the villi and increasing the activity of brush border cells, thereby potentially improving the digestion and absorption of nutrients [33]. Regarding the EPEF, incorporating zeolite into the diet can lead to a significantly positive impact on the performance and health of birds. This is evidenced by the improvements observed in EPEF. The EPEF results in the present study demonstrated efficient production, as the values were notably elevated compared to those in other studies [7, 35].

Any improvement in the intestinal environment is positively reflected in the health of birds [36]. Zeolites slow down the passage rate of digesta through the digestive tract and control the release of nutrients in the small intestine [11]. In the current study, the addition of dietary zeolite significantly reduced intestinal viscosity compared with the control group. Similarly, Ouhida et al. [37] indicated that the inclusion of sepiolite (clay minerals) and enzymes (β-glucanase and arabinoxylanases) could potentially decrease the viscosity of the intestine by forming a gel-like substance, which in turn could slow down the passage rate of digesta. This prolonged transit time may enhance the effectiveness of endogenous enzyme activity in breaking down proteins, carbohydrates, and fats, leading to improved nutrient digestibility. Birds fed 1.5% and 3% zeolite exhibited lower pH in the intestinal sections compared to the control group. This alteration could be attributed to the ion-exchange properties of zeolite, which might induce shifts in pH and ionic composition of gastrointestinal fluids, potentially leading to increased secretion of digestive enzymes [30]. Moreover, zeolites could stabilize gastrointestinal pH, creating a favorable environment for beneficial microorganisms essential for digestion and gut health. Additionally, dietary zeolite influences gut morphology, lowers pH, and reduces levels of pathogenic bacteria, indicating its potential for enhancing intestinal health [17, 30]. The function and natural structure of the intestine are the biological bases of growth, digestion, and absorption of nutrients in birds [38].

Actually, clay minerals (zeolite, montmorillonite, or kaolin) have been reported to be used as alternatives or complements for the use of antimicrobials in broiler feed [39]. Boosting the microbial environment in the intestines of birds is a major factor that affects the health status of birds, and this is what was demonstrated by zeolite feeding. The supplementation of diets with zeolite can yield positive impacts on the digestive environment by decreasing the counts of *E. coli* and total anaerobes. This contributes to the establishment of a balanced oxidized-reduced environment, ultimately leading to a reduction in anaerobic activity. Additionally, the addition of zeolite enhanced the populations of beneficial bacteria, such as *Lactobacillus*. Similar results were clarified by Qu et al. [19]. This effect may be attributed to the fact that zeolite had a comparable impact to antibiotics, effectively countering the growth of gram-negative bacteria (*E. coli* and anaerobes) [18]. Prasai et al. [32] showed the ability of zeolite to specifically decrease the prevalence of significant poultry zoonotic pathogens while maintaining the diversity of the chicken microbiota and minimizing substantial alterations in the gut microbial community. Finally, the use of zeolite presents a promising approach for substituting antibiotics in the poultry sector. This strategy offers the potential to enhance pathogen control, while maintaining the microbial balance.

Alterations in growth performance coincide with shifts in the secretion of digestive enzymes and nutrient digestibility [31, 40]. The addition of dietary zeolite significantly increased amylase activity. However, the activity of other digestive enzymes showed a rise as the dietary zeolite levels were elevated. This finding was in agreement with Zhou et al. [14], who concluded that supplementing with a 2% combination of zeolite and attapulgite significantly increased lipase, amylase, and trypsin levels. Wu et al. [31] noted that supplementing diets of broiler chicks with clinoptilolite had positive impacts on gut development and led to significant increases in the activities of digestive enzymes such as chymotrypsin, protease, amylase, and trypsin in the contents of the small intestine. Finally, zeolite can contribute to an increase in digestibility and feed utilization.

Although the addition of zeolite resulted in improved body weight, it was not reflected in the carcass traits. These findings are consistent with the conclusions reported by Schneider et al. [9], indicating that the inclusion of dietary zeolite does not lead to an improvement in broiler carcass%. On the contrary, carcass percentage exhibited a significant impact from the addition of dietary zeolite, as reported by Emam et al. [12]. Regarding the gizzard%, birds fed dietary zeolite had higher values compared with the control group; this may be due to the fact that feeding on zeolite could be the reason for the increase in gizzard weight.

Ammonia release from poultry farms is a concerning issue as it is a pollutant that can harm the well-being of birds [41]. The inclusion of dietary zeolite led to a significant decrease in blood ammonia levels of broiler birds. Emam et al. [8] stated that the treatment with zeolite can reduce serum ammonia levels of birds. This effect can be associated with zeolite’s active adsorption capacity for carbon dioxide, ammonia, mercaptans, and hydrogen sulfide, resulting in the elimination of toxins and improvements in immunological responses [17] Additionally, this impact could potentially result from the large surface area of zeolite, which enhances its adsorption properties. The reduction in blood ammonia levels would improve the status of the respiratory system of birds.

Birds that were fed a dietary zeolite exhibited a significant increase in albumin and total protein concentrations compared to the control group. This increase might be attributed to zeolite’s potential to enhance liver function, consequently leading to a rise in both total protein and albumin levels, which is also reflected in the elevated levels of ALT and AST in these birds. Similarly, Emam et al. [12] revealed significant differences in albumin levels in broilers fed with dietary zeolite, which were in accordance with the results of the present study. The inclusion of zeolite in the diets had no impact on the serum albumin and total protein of broiler chickens [15]. Similarly, Pavlak et al. [16] reported that the inclusion of zeolite at 5,000 and 10,000 g/ton did not alter serum total protein, albumin, ALT, and AST in the birds at 42 days of age. With an increase in the dietary zeolite level, there was a significant reduction in serum total bilirubin, uric acid and creatinine levels. This decline suggests an improvement in hepatic and renal function in the treated birds. Additionally, this decrease may be attributed to the detoxification mechanisms and remarkable absorption capacity of zeolite [42]. Similarly, Emam et al. [12] reported that supplementing diets with 3% and 6% zeolite led to a decrease in creatinine levels in broiler chicks exposed to saline well water, compared to the untreated birds. The elevated concentrations of serum calcium and phosphorus could potentially be attributed to the augmented zeolite inclusion in the diet. The favorable impacts of zeolite might be linked to its silicon, aluminum or sodium content, as these minerals have been shown to influence calcium metabolism, thereby enhancing phosphorus and calcium utilization [43]. Therefore, the characteristics of the zeolite and its mineral content play a vital role in increasing the serum phosphorus and calcium levels of the treated birds. Eleroğlu et al. [44] stated a positive impact of zeolite on maintaining a favorable balance of serum calcium and phosphorus levels, as well as its role in regulating electrolyte balance in birds. This could potentially result in an increase in blood levels of calcium and phosphorus. The selective absorption mechanism helps to optimize mineral uptake, reduce competition with other minerals, and improve overall nutrient status [45, 46].

In the current study, the dietary zeolite at different levels was found to be effective in enhancing hematological parameters. Similarly, Emam et al. [12] stated that adding 3% and 6% zeolite to the diets significantly enhanced the chickens’ hematological parameters (RBCs, Hb and HCT). This improvement implies that zeolite likely plays a vital role in protecting the birds by reducing toxic substances like heavy metal salts [42]. In a similar trend, Al-Musawy et al. [13] revealed that supplementing broiler chicken diets with zeolite at varying levels of 1, 2 and 3% notably increased RBCs and Hb concentrations. Overall, zeolite supplementation seems to positively influence the chickens’ well-being when facing challenging conditions. In the present study, adding zeolite to broiler diets led to an increase in serum iron and a decrease in TIBC. Iron is a vital component of Hb that is the oxygen-carrying protein in RBCs [47]. Following zeolite supplementation, serum iron concentration was increased compared with the control, resulting in improvement of hematological parameters and subsequently health status of birds. Emam et al. [8] found that adding natural zeolite to the litter significantly increased serum iron and was associated with a reduction in TIBC for the zeolite-treated group compared with the control group. Zeolites may improve iron profile by removing substances that hinder its uptake and potentially influencing iron absorption in the digestive tract.

Antioxidant status is an important consideration in studying the effect of any natural substance added to feed on poultry [48, 49]. In the present results, the zeolite-treated groups displayed higher values of antioxidant indices compared to the control group. These results indicate that the inclusion of zeolite could enhance the antioxidant system. These results align with the observations of Zhou et al. [14], who indicated that the increased antioxidant potential of the zeolite and attapulgite (a hydrated magnesium aluminum silicate) combination can be attributed to their antibacterial properties. These substances have the ability to adsorb harmful bacteria that are vulnerable to oxidative stress, consequently enhancing the overall antioxidant influences of the combined mixture. Moreover, the combined zeolite and attapulgite mixture also could adsorb free radicals, which are known to play a role in tissue damage. Qu et al. [19] studied the effects of dietary zeolite as an antibiotic alternative, and found that intestinal antioxidant enzyme activities (T-AOC and SOD) were similar among groups.

## Conclusions

The findings indicated that the inclusion of zeolite at levels of 1.5–3.0% in broiler diets had positive effects on various parameters including iron profile, blood ammonia, intestinal pH, and viscosity, cecal microbiota, performance, and blood constituents. This demonstrates its potential as a beneficial dietary supplement to improve broiler production. This requires further research into its mode of action, dose-response relationship, and possible synergistic effects with other feed additives.

## Data Availability

The datasets used and analyzed during the current study are available from the corresponding author upon reasonable request.
